# Attenuation and Stability of CHIKV-NoLS, a Live-Attenuated Chikungunya Virus Vaccine Candidate

**DOI:** 10.3390/vaccines7010002

**Published:** 2018-12-22

**Authors:** Eranga Abeyratne, Joseph R. Freitas, Ali Zaid, Suresh Mahalingam, Adam Taylor

**Affiliations:** Emerging Viruses and Inflammation Research Group, Institute for Glycomics, Griffith University, Gold Coast, QLD 4222, Australia; e.abeyratne@griffith.edu.au (E.A.); j.freitas@griffith.edu.au (J.R.F.); a.zaid@griffith.edu.au (A.Z.); s.mahalingam@griffith.edu.au (S.M.)

**Keywords:** alphavirus, chikungunya virus, live-attenuated, vaccine, preclinical

## Abstract

Our previous investigation of the nucleolar localisation sequence (NoLS) of chikungunya virus (CHIKV) capsid protein demonstrated the role of capsid in CHIKV virulence. Mutating the NoLS of capsid in CHIKV led to the development of a unique live-attenuated CHIKV vaccine candidate, termed CHIKV-NoLS. CHIKV-NoLS-immunised mice developed long-term immunity from CHIKV infection after a single dose. To further evaluate CHIKV-NoLS attenuation and suitability as a vaccine, we examined the footpad of inoculated mice for underlying CHIKV-NoLS-induced immunopathology by histological and flow cytometric analysis. In comparison to CHIKV-WT-infected mice, CHIKV-NoLS-inoculated mice exhibited minimal inflammation and tissue damage. To examine the stability of attenuation, the plaque phenotype and replication kinetics of CHIKV-NoLS were determined following extended in vitro passage. The average plaque size of CHIKV-NoLS remained notably smaller than CHIKV-WT after extended passage and attenuated replication was maintained. To examine thermostability, CHIKV-NoLS was stored at 21 °C, 4 °C, −20 °C and −80 °C and infectious CHIKV-NoLS quantified up to 84 days. The infectious titre of CHIKV-NoLS remains stable after 56 days when stored at either −20 °C or −80 °C. Interestingly, unlike CHIKV-WT, the infectious titre of CHIKV-NoLS is not sensitive to freeze thaw cycles. These data further demonstrate preclinical safety and stability of CHIKV-NoLS.

## 1. Introduction

Chikungunya virus (CHIKV) is an arthropod-borne virus transmitted to humans by mosquitoes, principally of the genus *Aedes*. CHIKV belongs to the genus *Alphavirus* of the *Togaviridae* family and has a positive sense, single strand RNA genome of 11.8 kb that consists of two open reading frames (ORFs), the non-structural protein and the structural protein ORFs [1,2]. Chikungunya virus disease (CHIKVD) in patients commonly manifests as high fever, severe joint pain, maculopapular rash, polyarthralgia, myalgia and edema of extremities. CHIKV is associated with a high rate of symptomatic infection and clinical disease is mostly self-limiting [3]. However, a proportion of individuals experience chronic polyarthritis or relapses of joint pain for months to years after the initial infection [4]. Rare cases of CHIKVD have also reported neurological complications [5].

A sylvatic transmission cycle has been demonstrated in non-human primates, which are thought to act as the primary reservoir for CHIKV [6]. *Aedes aegypti* and *Aedes albopictus* species are thought to be largely responsible for the urban cycle of human–mosquito–human CHIKV transmission. Massive epidemics of CHIKVD occurred in Indian Ocean Islands, South Asian and Southeast Asian countries following the Kenyan outbreak of CHIKV in 2004 [7,8,9]. In this period CHIKV was introduced to many territories where it had not been found previously. Approximately 300,000 cases of CHIKVD were suspected in the La Reunion Island outbreak in 2006, accounting for a third of the island’s population [7]. In 2013 CHIKV reached the western hemisphere with outbreaks in Islands of the Caribbean, North, Central and South American countries. CHIKV reached 45 countries in the Americas outbreak affecting approximately 2 million people [10]. In 2006 the cost incurred due to direct and indirect health expenses during La Reunion outbreak was estimated to be €43.9 million [11]. The economic burden of disease during the 2014 outbreak in Colombia stretched to around US $73.5 million [12].

The rapid re-emergence of CHIKV can be partly due to socio-economic factors such as increased number of overseas travellers, changes in land use and urbanisation with high population densities. Changes in geographic distribution of mosquito vectors and vector competence have also played an important role in these decade long outbreaks [13,14]. Patient and outbreak management during CHIKV epidemics mainly focuses on control of mosquito vectors and management of clinical cases with symptomatic therapy. To date there are no commercially available antivirals or vaccines to protect against CHIKV infection. Since the antigenic diversity exhibited by CHIKV is limited and the virus is incapable of re-infection, a vaccine can be the best solution to counteract disease and prevent circulation amongst populations.

We have developed a unique live-attenuated CHIKV vaccine candidate named CHIKV-NoLS. Mutating the nucleolar localisation sequence (NoLS) of capsid protein by replacing ten wild-type (WT) amino acids with alanines was found to significantly attenuate virus replication [15]. CHIKV-NoLS produced a small plaque phenotype, again suggestive of attenuation and an inability to spread from the initial site of infection. CHIKV-NoLS-inoculated mice showed no signs of disease and developed long-term immunity from CHIKV-WT challenge with a single dose of vaccine candidate [15]. Here we expand our studies with CHIKV-NoLS to further test its attenuation, stability and suitability for further preclinical development.

## 2. Materials and Methods 

### 2.1. Viruses and Cells

The CHIKV-NoLS infectious clone was produced as outlined previously [15]. Viruses were rescued from infectious clones by linearization of the plasmid and transcription of the viral genomic RNA using the mMESSAGE mMACHINE™ SP6 Transcription Kit (Thermo Fisher, Brisbane, Australia). RNA was transfected into Vero cells using Lipofectamine 2000 Transfection Reagent according to the manufacturer’s instructions (Thermo Fisher, Australia) and virus was harvested 36–48 h later. CHIKV-NoLS derived from transfected cells (passage 0) was used without further passage unless described otherwise. Vero cells were maintained at 37 °C with 5% CO_2_ in Dulbecco’s Modified Eagle’s Medium (DMEM) supplemented with 10% fetal calf serum (FCS). C6/36 cells were cultured in Leibovitz’s L-15 medium (Thermo Fisher), supplemented with 10% tryptose phosphate broth and 10% FCS.

### 2.2. Mice

C57BL/6 WT mice were obtained from the Animal Resources Centre (Perth, Australia). Groups of 5 twenty-eight-day-old C57BL/6 male and female mice, in equal distribution, were inoculated in the ventral/lateral side of the right foot with 10^4^ plaque forming units (PFU) CHIKV-WT or CHIKV-NoLS diluted in phosphate buffered saline (PBS) to a volume of 20 µL. Mock-infected mice were inoculated with PBS alone. Mice were weighed and scored for disease signs every 24 h and sacrificed by CO_2_ asphyxiation at experimental endpoints. CHIKV-induced footpad swelling was assessed by measuring the height and width of the perimetatarsal area of the hind foot, using Kincrome digital vernier calipers. All animal procedures and experiments were performed in accordance with the guidelines set out by The Griffith University Animal Ethics Committee (Ethics Number GLY/05/15). 

### 2.3. Histology

Mice were euthanized at experimental endpoints. Inoculated feet were dissected and fixed in 4% paraformaldehyde, followed by decalcification in 14% ethylenediaminetetra-acetic acid and paraffin embedding. Sections 5 μm thick were prepared and stained with hematoxylin and eosin (H&E). Images were taken on a Nikon Eclipse TS100 and are representative of 5 mice per group. Images are representative of at least 6 fields of view and cellular infiltrates were quantified at 200× magnification using ImageJ software (National Institutes of Health, Bethesda, MD, USA).

### 2.4. Enumeration of Foot Cellular Infiltrates by Flow Cytometry

Mice were euthanized at experimental endpoints and perfused with ice-cold PBS. Inoculated feet were dissected and carefully degloved. Feet were added to Type III Collagenase (2 mg/mL; Worthington Biochemicals, Lakewood, NJ, USA) containing DNAse I (5 mg/mL; Sigma Aldrich, St. Louis, MO, USA) and dissociated for 1 h at 37 °C. Following digestion, foot tissue was vigorously mixed in RPMI with 10% FCS (RP10) and filtered through 70 µm cells strainer, and washed with RP10. Cells suspensions were then filtered through 30 µm, resuspended in staining buffer (PBS with 2% FCS; 5mM EDTA), blocked with anti-CD16/32 Fc block (93; BD Biosciences, Franklin Lakes, NJ, USA), and labelled with fluorochrome-conjugated antibodies against mouse CD45 (30-F11; eBiosciences), CD3 (17A2; Biolegend), CD4 (RM4-5; BD Biosciences), CD8 (53-6.7, BD Biosciences), CD11b (M1/70; BD Biosciences), Ly6G (1A8; BD Biosciences), Ly6C (HK 1.4; BD Biosciences), and IA/IE (clone M5/114; Biolegend, San Diego, CA, USA). Dead cells were stained using LIVE/DEAD NIR viability dye (Thermo Fisher). Counting beads (BD Spherotec; BD Biosciences) were added to samples before acquisition on a BD LSR II Fortessa. Data was analysed using FlowJo (v10.5.2; TreeStar Inc., Ashland, OR, USA). 

### 2.5. Extended in Vitro Passage

To examine the stability of CHIKV-NoLS attenuated replication CHIKV-NoLS was passaged ten times in Vero cells at 37 °C using a multiplicity of infection (MOI) of 0.1. C6/36 cells were infected with CHIKV-WT, passage zero CHIKV-NoLS or passage five CHIKV-NoLS at an MOI of 0.1 and allowed to incubate for 1 h at 28 °C in a 5% CO_2_ incubator. Virus was removed and the cells were washed with PBS and overlaid with Leibovitz’s L-15 medium, supplemented with 10% tryptose phosphate broth and 10% FCS. At the indicated time points supernatant aliquots were harvested and viral titres measured by plaque assay as outlined previously [15]. To examine the stability of the small plaque phenotype of CHIKV-NoLS, Vero cells were infected with 30 PFU of passaged CHIKV-NoLS. Virus was allowed to incubate for 1 h at 37 °C in a 5% CO_2_ incubator before the virus was removed and the cells overlaid with Opti-MEM containing 3% FCS and 1% agarose (Sigma-Aldrich) and incubated for 48 h in a 5% CO_2_ incubator. Cells were fixed in 1% formalin, and virus plaques were made visible by staining with 0.1% crystal violet. Plaque size was measured using ImageJ software. Plaque size of all passaged virus was examined at one time to remove the potential for variation of plaque size between assays.

### 2.6. Thermostability Assays

The thermostability of CHIKV-NoLS was compared to CHIKV-WT. Viruses were diluted to 5 × 10^5^ PFU/mL and vials stored at 21 °C, 4 °C, −20 °C and −80 °C. At the indicated time points infectious CHIKV-NoLS and CHIKV-WT was quantified by plaque assay, as reported previously [15]. To examine the effect of repeated freeze thaw cycles on viablility of infectious CHIKV-NoLS, viruses stored at −80 °C were thawed fully on ice. Viral titres were measured by plaque assay as outlined previously [15]. Following thawing on ice, virus was returned to −80 °C and stored at −80 °C for no less than 1 day and no longer than 3 days before repeating thawing.

### 2.7. Statistical Analysis

Statistical significance of differences between groups from histological analysis was assessed using one-way analysis of variance (ANOVA) with Tukey’s post-test. Images are representative of at least 6 fields of view from 5 mice per group. Statistical significance of differences between groups from flow cytometry analysis was assessed using either a non-parametric Mann–Whitney U-test, or a two-way ANOVA with a Holm-Sidak comparison correction. Statistical significance of differences between multistep growth kinetics was obtained by two-way ANOVA with Bonferroni post-tests. Differences where *p* <0.05 were considered statistically significant. Biological replicates were analysed from 3 independent experiments.

## 3. Results

### 3.1. Immunopathology Associated with CHIKV-NoLS Inoculation 

**Figure 1:**
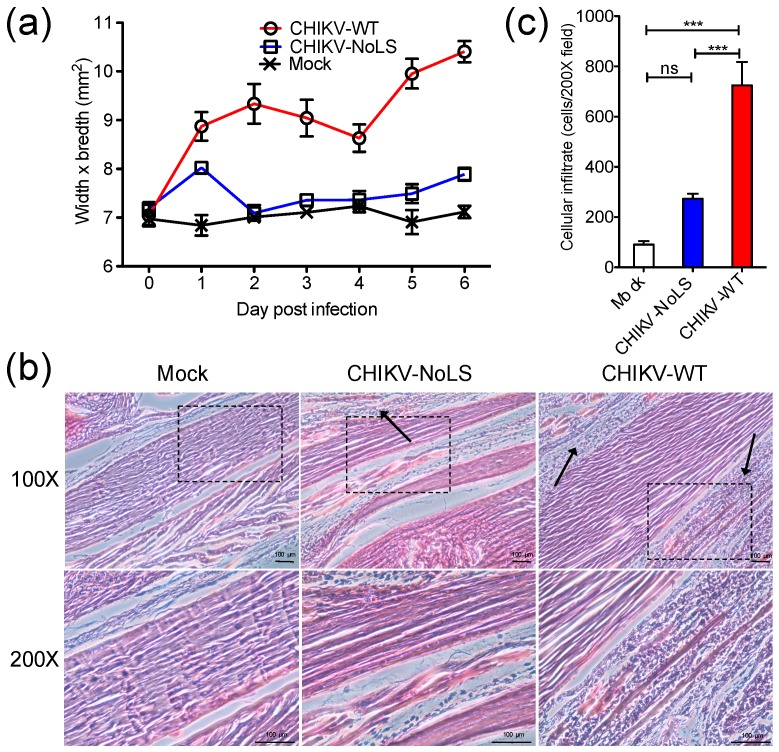
Histological analysis of the mouse footpad after inoculation with 10^4^ plaque forming units (PFU) CHIKV-WT or CHIKV-NoLS. Twenty-eight-day-old C57BL/6 mice were inoculated in the ventral/lateral side of the right foot with 10^4^ PFU CHIKV-WT or CHIKV-NoLS or mock-infected with (phosphate buffered saline) PBS alone. (**a**) CHIKV-induced footpad swelling was assessed daily by measuring the height and width of the perimetatarsal area of the hind foot. Each symbol represents the mean ± standard error from 5 mice; (**b**) Inoculated (ipsilateral) feet were dissected, processed for histological analysis and stained with H&E. Images at 100× and 200× magnification are representative of at least 6 fields of view. Black arrows indicate cellular infiltration. The black dashed box indicates the area magnified at 200×. The size bars represent 100 µm; (**c**) Cellular infiltrates at 200× magnification were quantified using ImageJ software. ***, *p* < 0.001, ns―not significant. Statistical analyses were performed using one-way ANOVA with Tukey’s post-test. Data represents the mean ± standard error.

Using the C57BL/6 mouse model of CHIKVD, which measures swelling of the perimetatarsal area of the inoculated hind foot, we demonstrated that CHIKV-NoLS-inoculated mice present with no disease signs [15]. Histological and flow cytometric analysis was performed on the feet of mice to examine the underlying pathology and immunopathology associated with CHIKV-NoLS inoculation. C57BL/6 mice were inoculated in the ventral/lateral side of the right foot with 10^4^ PFU CHIKV-WT or CHIKV-NoLS or mock-infected with PBS. Footpad swelling was measured (Figure 1a) and tissues were harvested on day 6 post inoculation and processed for histology and flow cytometry. CHIKV-NoLS and mock-inoculated mice showed no signs of disease. Histopathological findings show that CHIKV-WT-infected mice had extensive cellularity in the footpad (Figure 1b). Compared to CHIKV-WT, inflammation in the footpad of CHIKV-NoLS-inoculated mice was unremarkable with minimal cellular infiltration (Figure 1c).

**Figure 2:**
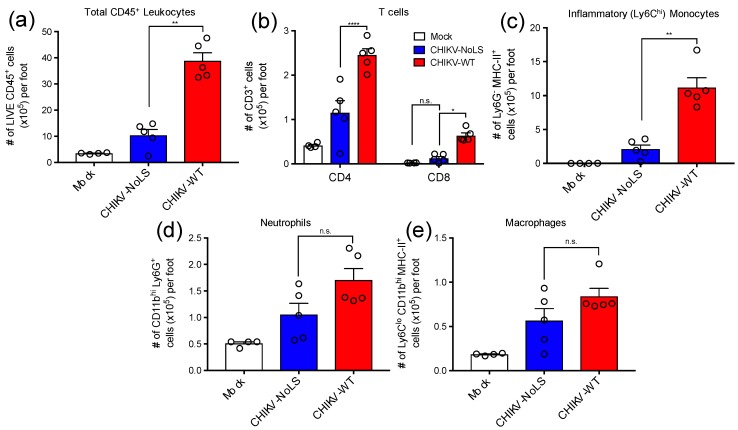
Cellular infiltrates in the feet of inoculated mice were analysed using flow cytometry. (**a**) Total live CD45^+^ leukocytes, (**b**) CD4^+^ and CD8^+^ T cells (CD3^+^), (**c**) inflammatory monocytes (Ly6C^hi^ Ly6G^-^ CD11b^hi^ MHC-II^+^), (**d**) Neutrophils (Ly6G^hi^ CD11b^hi^) and (**e**) tissue macrophages (Ly6C^lo^ CD11b^hi^ Ly6G^-^ MHC-II^+^). *, *p* < 0.05, **, *p* < 0.01, and ****, *p* < 0.0001. Statistical analyses were performed using a Mann–Whitney U-test (A, B, C, D) or a a two-way ANOVA with a Holm-Sidak comparison correction. Data represents the mean ± standard error.

CD4^+^ T cells have a pathogenic role in CHIKV disease and are thought to be directly responsible for joint swelling [16]. To assess the composition of immune cell infiltrates associated with CHIKV-NoLS inoculation, feet of mice inoculated with CHIKV-WT, CHIKV-NoLS or mock were processed for flow cytometry analysis. The number of total CD45^+^ leukocytes was approximately 4-fold higher in the feet of CHIKV-WT-infected mice compared to that of CHIKV-NoLS-inoculated mice (Figure 2a). Interestingly, the number of CD4^+^ and CD8^+^ T cells was significantly lower in the feet of mice inoculated with CHIKV-NoLS compared to CHIKV-WT (Figure 2b). In addition, the number of myeloid cells such as Ly6C^hi^ inflammatory monocytes, which constituted the majority of this immune cell infiltrate, was significantly lower in the feet of CHIKV-NoLS-inoculated mice (Figure 2c) compared to CHIKV-WT. Further, the number of Ly6G^+^ neutrophils (Figure 2d) and MHC-II^+^ Ly6C^lo^ macrophages (Figure 2e) were also reduced, though not significantly, in the feet of CHIKV-NoLS-inoculated mice. Overall, data indicates that CHIKV-NoLS inoculation is associated with a reduction in cellular infiltrates compared to CHIKV-WT, and that T cells and inflammatory monocytes are the main immune cell subsets implicated in this local inflammatory response following CHIKV infection.

### 3.2. Stability of CHIKV-NoLS Attenuation Following Extended in Vitro Passage

**Figure 3:**
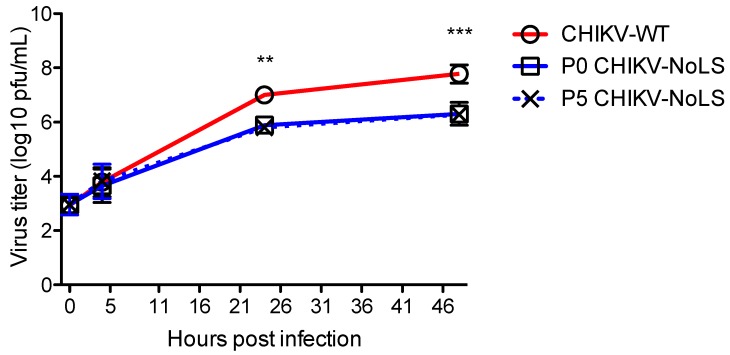
Multistep growth kinetics of CHIKV-NoLS in C6/36 cells after extended in vitro passage. C6/36 cells were infected with CHIKV-WT, P0 CHIKV-NoLS or P5 CHIKV-NoLS at an MOI of 0.1 PFU/cell. Supernatants were collected at the indicated time points, and infectious virus was quantified by plaque assay. **, *p* < 0.01, and ***, *p* < 0.001. Statistical analyses were performed using two-way ANOVA with Bonferroni post-tests. Each symbol represents the mean ± standard error from 3 independent experiments.

Attenuated replication of CHIKV-NoLS in mosquito (*Aedes albopictus*)-derived C6/36 cells may prevent infection of mosquito vectors, avoiding unwanted transmission of the vaccine [15]. To assess the stability of CHIKV-NoLS attenuation, extended in vitro passage of CHIKV-NoLS was performed five times in Vero cells and the replication kinetics of CHIKV-NoLS (P0) and passaged CHIKV-NoLS (P5) compared to CHIKV-WT in C6/36 cells. Multistep growth kinetics in C6/36 cells was obtained by inoculating cells at an MOI of 0.1 PFU. Supernatants were collected at the indicated time points and infectious particles quantified by plaque assay. P5 CHIKV-NoLS shows similar multistep replication kinetics to P0 CHIKV-NoLS (Figure 3). P0 CHIKV-NoLS and P5 CHIKV-NoLS show significantly reduced infectious titres at 24 and 48 hours post inoculation compared to CHIKV-WT (Figure 3). To examine the genetic stability of CHIKV-NoLS after extended in vitro passage, three plaques of P5 CHIKV-NoLS were plaque purified and Sanger sequenced. Two of the P5 CHIKV-NoLS contained no mutations in capsid protein. The third P5 CHIKV-NoLS acquired one synonymous nucleotide change in capsid protein that was within the NoLS mutation, a A101S substitution. This substitution did not change the small plaque phenotype or attenuated replication kinetics of CHIKV-NoLS. Results indicate that after five passages in Vero cells, replication of CHIKV-NoLS in insect cells remains significantly impaired compared to CHIKV-WT. The attenuated replication kinetics of CHIKV-NoLS remains stable after five passages in vitro.

**Figure 4:**
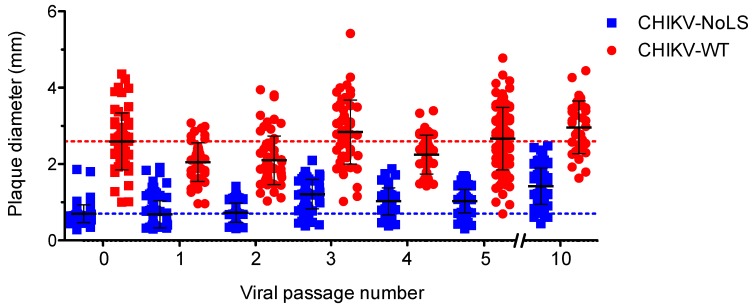
CHIKV-NoLS plaque size after extended in vitro passage. CHIKV-NoLS and CHIKV-WT were passaged 10 times in Vero cells at 37 °C using a multiplicity of infection of 0.1 PFU/cell. With each passage of virus, Vero cells were infected with 30 PFU and plaque size measured using ImageJ software. Each dot represents one plaque. Dashed lines represent the mean plaque size for CHIKV-NoLS and CHIKV-WT at passage 0.

CHIKV-NoLS has a small plaque phenotype compared to CHIKV-WT [15]. Small plaques indicate a reduced ability of the vaccine to spread from the initial site of infection and are thus a useful marker of CHIKV-NoLS attenuation. To examine the phenotypic stability of CHIKV-NoLS attenuation extended in vitro passage of CHIKV-NoLS and CHIKV-WT was performed ten times in Vero cells. The average plaque size of CHIKV-NoLS remained notably smaller than CHIKV-WT after ten passages (Figure 4). CHIKV-NoLS also exhibited more homogeneous plaque morphology than CHIKV-WT. Results show that, after ten passages, CHIKV-NoLS does not revert to a CHIKV-WT plaque phenotype and suggest that the CHIKV-NoLS vaccine candidate remains attenuated in its ability to spread from the initial site of infection.

### 3.3. Examining the Thermotability of CHIKV-NoLS 

**Figure 5:**
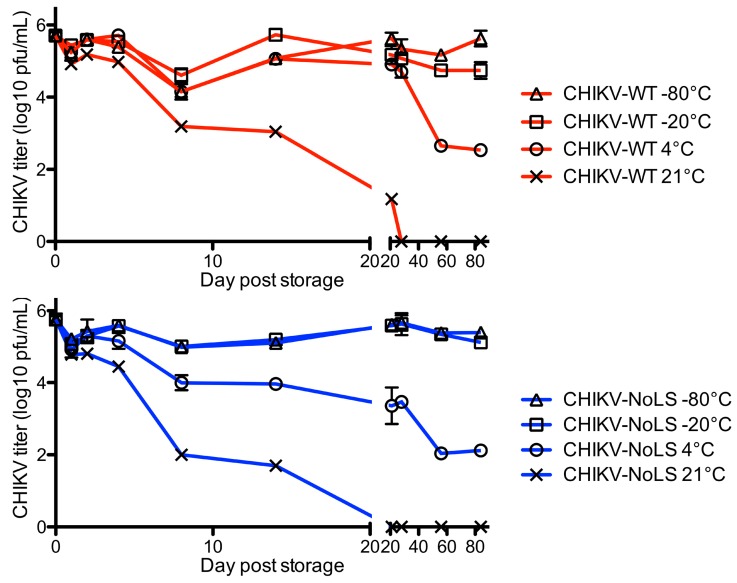
Long term thermostability of CHIKV-NoLS. CHIKV-NoLS and CHIKV-WT were diluted to 5 × 10^5^ pfu/ml and vials stored at 21 °C, 4 °C, −20 °C and −80 °C. At the indicated time points infectious CHIKV-NoLS and CHIKV-WT was quantified by plaque assay. Each symbol represents the mean ± standard error.

Although able to be stored stably at −80 °C, storage of CHIKV-NoLS at −20 °C or 4 °C would increase the accessibility of CHIKV-NoLS to vulnerable populations in CHIKV endemic areas and reduce the costs associated with storage. CHIKV-NoLS thermostability would be advantageous to further vaccine preclinical development. To investigate vaccine thermostability, CHIKV-NoLS and CHIKV-WT were diluted to 5 × 10^5^ PFU/mL and vials stored at 21 °C, 4 °C, −20 °C and −80 °C. At the indicated time points infectious CHIKV-NoLS and CHIKV-WT were quantified by plaque assay. Infectivity of CHIKV-NoLS and CHIKV-WT dramatically reduced when stored at 21 °C, room temperature (Figure 5). No infectious CHIKV-NoLS or CHIKV-WT was detected after 28 days when stored at 21 °C. After 56 days at 4 °C the titre of CHIKV-NoLS fell to 110 PFU/mL and CHIKV-WT to 450 PFU/mL. The titre of CHIKV-NoLS and CHIKV-WT remained stable after 56 days when stored at either −20 °C or −80 °C.

**Figure 6:**
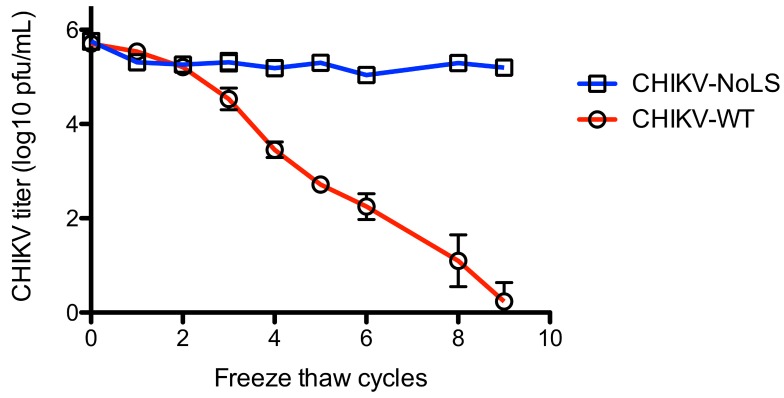
Stability of CHIKV-NoLS infectivity following repeated freeze thaw cycles. CHIKV-NoLS and CHIKV-WT were diluted to 5 × 10^5^ PFU/mL and vials stored at −80 °C. Vials were thawed on ice and infectious CHIKV-NoLS and CHIKV-WT quantified by plaque assay. Vials were returned to −80 °C and the process repeated. Each symbol represents the mean ± standard error.

To further examine the stability of CHIKV-NoLS, infectivity was measured after freeze thaw. Vials of CHIKV-NoLS and CHIKV-WT at 5 × 10^5^ PFU/mL were subjected to nine freeze thaw cycles. After each thaw infectious CHIKV-NoLS and CHIKV-WT was quantified by plaque assay. CHIKV-WT infectivity decreased with every freeze thaw cycle (Figure 6). After nine freeze thaw cycles the infectivity of CHIKV-WT was just above the limit of detection. Interestingly, CHIKV-NoLS saw no loss in infectivity after freeze thaw (Figure 6). Overall, CHIKV-NoLS showed good stability when stored at −20 °C and −80 °C for up to 56 days and compared to CHIKV-WT was resistant to loss of infectivity due to freeze–thaw.

## 4. Discussion

A live-attenuated CHIKV vaccine candidate is a desirable means of protecting against CHIKV infection due to swift and long-lasting immunogenicity following a single dose immunisation. This fast acting, one shot approach is ideally suited to CHIKV outbreak control where infection can spread rapidly. Failure of patients to return for second and third immunisations is a concern for vaccine candidates that require booster regimes to achieve seroconversion. Additionally, prime-boost immunisation studies against CHIKV have shown that sub-neutralising levels of antibody induced by priming only may lead to enhancement of inflammation [17]. Antibody-mediated enhancement of CHIKV infection has been shown to aggravate CHIKVD severity [18]. Vaccine strategies that require multiple doses to elicit a protective immune response may need to consider how to avoid antibody-mediated enhancement of CHIKV infection induced by vaccination [18]. A vaccine, such as CHIKV-NoLS, that is sufficiently immunogenic to provide protection after one shot eliminates the need for follow-up vaccination and the potential for antibody-mediated enhancement.

An early live-attenuated CHIKV vaccine candidate has proven to be safe, produce well-tolerated side effects, and be highly immunogenic in phase II human trials after a single immunisation [19]. However, reactogenicity in a number of vaccinees halted development of this vaccine candidate. With attenuation mediated by only two point mutations, the risk of reversion to WT also raised safety concerns over this vaccine. CHIKV-NoLS has previously demonstrated immunogenicity and a high level of attenuation with no disease signs, as well as baseline levels of CHIKVD pro-inflammatory factors in inoculated C57BL/6 mice [15]. Despite the lack of disease signs in inoculated mice, to further preclinical development of CHIKV-NoLS it is important to understand the stability of CHIKV-NoLS attenuation and underlying pathology associated with vaccine inoculation.

To examine the immunopathology associated with CHIKV-NoLS inoculation, histological and flow cytometry analysis was performed on the feet of inoculated mice. Mice inoculated with CHIKV-NoLS showed minimal inflammation and significantly reduced cellular infiltration compared to CHIKV-WT-infected mice. Interestingly, CD4^+^ T cells and inflammatory monocytes, independently identified as mediators of CHIKV pathogenesis, were significantly reduced in CHIKV-NoLS-inoculated mice compared to CHIKV-WT-infected mice [16,20]. The reduced migration of multiple cellular mediators of CHIKVD in CHIKV-NoLS-inoculated mice is evidence of the extent to which CHIKV-NoLS is attenuated and further implicates CD4^+^ T cells and inflammatory monocytes in CHIKV pathogenesis.

Although our previous studies demonstrate that the reduced replicative ability of CHIKV-NoLS impedes productive replication in vivo, we evaluated the stability of CHIKV-NoLS attenuation following extended in vitro passage [15]. After five serial passages the attenuated replication kinetics of CHIKV-NoLS remained unchanged in mosquito-derived C6/36 cells. Of three CHIKV-NoLS plaques purified and sequenced following extended in vitro passage only one acquired a synonymous nucleotide change in capsid protein, a A101S substitution within the NoLS mutation. As the WT amino acid at this position is arginine the impact of this substitution on capsid function is unclear. Interestingly, the small plaque phenotype, a hallmark of an inability of CHIKV-NoLS to successfully propagate, was observed for up to ten passages. CHIKV-NoLS plaques remained notably smaller and more homogeneous than CHIKV-WT plaques. The stability of attenuation is critical to the development of live-attenuated vaccine candidates. The thermostability of CHIKV-NoLS remained stable after 56 days when stored at either −20 °C or −80 °C. Alternative approaches to improve the stability of CHIKV-NoLS at higher temperatures and thus increase the accessibility of the vaccine candidate to target populations are currently under investigation. Lyophilisation has been used to successfully stabilize numerous live-attenuated vaccine candidates [21]. The development of a lyophilisation strategy for CHIKV-NoLS and its effect on CHIKV-NoLS vaccine potency warrants further investigation.

Although it is clear that the NoLS mutation disrupts virus replication downstream of viral RNA production, the exact mechanism of virus attenuation remains unclear [15]. The NoLS mutation is a large alteration of a highly basic capsid region (two nucleotide changes in 10 codons). As capsid is a multifunctional structural protein it is possible that the NoLS mutation disrupts a number of capsid protein functions. For example, the NoLS mutation is close to motifs implicated in RNA binding, nucleocapsid assembly and capsid interaction with the envelope proteins [22]. It follows that structural changes in the CHIKV-NoLS particle may be responsible for the increased resistance to freeze thaw.

## 5. Conclusions

These data further demonstrate that CHIKV-NoLS is highly attenuated in vivo and that the vaccine candidate remains stably attenuated following extended in vitro passage. Results support the preclinical development of CHIKV-NoLS with the aim to develop a vaccine candidate able to meet the need to control explosive large-scale outbreaks of CHIKV.

## 6. Patents

S.M. and A.T. are inventors on a new international (PCT) patent application (PCT/AU2017/050489) for the vaccine candidate CHIKV-NoLS.
